# Deep brain stimulation in Parkinson’s disease: A scientometric and bibliometric analysis, trends, and research hotspots

**DOI:** 10.1097/MD.0000000000038152

**Published:** 2024-05-17

**Authors:** Vinay Suresh, Tirth Dave, Shankhaneel Ghosh, Rahul Jena, Vivek Sanker

**Affiliations:** a King George’s Medical University, Lucknow, India; b Bukovinian State Medical University, Chernivtsi, Ukraine; c Institute of Medical Sciences and SUM Hospital, Bhubaneshwar, India; d Bharati Vidyapeeth Medical College, Pune, India; e Society of Brain Mapping and Therapeutics, Los Angeles, CA.

**Keywords:** bibliometric analysis, DBS, deep brain stimulation, Parkinson disease

## Abstract

Parkinson disease (PD), a prevalent neurodegenerative ailment in the elderly, relies mainly on pharmacotherapy, yet deep brain stimulation (DBS) emerges as a vital remedy for refractory cases. This study performs a bibliometric analysis on DBS in PD, delving into research trends and study impact to offer comprehensive insights for researchers, clinicians, and policymakers, illuminating the current state and evolutionary trajectory of research in this domain. A systematic search on March 13, 2023, in the Scopus database utilized keywords like “Parkinson disease,” “PD,” “Parkinsonism,” “Deep brain stimulation,” and “DBS.” The top 1000 highly cited publications on DBS in PD underwent scientometric analysis via VOS Viewer and R Studio’s Bibliometrix package, covering publication characteristics, co-authorship, keyword co-occurrence, thematic clustering, and trend topics. The bibliometric analysis spanned 1984 to 2021, involving 1000 cited articles from 202 sources. The average number of citations per document were 140.9, with 31,854 references. “Movement Disorders” led in publications (n = 98), followed by “Brain” (n = 78) and “Neurology” (n = 65). The University of Oxford featured prominently. Thematic keyword clustering identified 9 core research areas, such as neuropsychological function and motor circuit electrophysiology. The shift from historical neurosurgical procedures to contemporary focuses like “beta oscillations” and “neuroethics” was evident. The bibliometric analysis emphasizes UK and US dominance, outlining 9 key research areas pivotal for reshaping Parkinson treatment. A discernible shift from invasive neurosurgery to DBS is observed. The call for personalized DBS, integration with NIBS, and exploration of innovative avenues marks the trajectory for future research.

## 1. Introduction

Parkinson disease (PD) is the second most prevalent neurodegenerative condition, affecting 2% to 3% of people over 65 years of age.^[1]^ PD is neuropathologically characterized by the loss of neurons in the substantia nigra, resulting in striatal dopamine deficits and intracellular inclusions containing aggregates of synuclein. PD is typically diagnosed when motor symptoms, such as bradykinesia, rigidity, and tremor, develop. Non-motor symptoms can be present at varying levels in all stages of Parkinson, although they tend to become more noticeable and standard as the disease progresses. Dementia, cognitive decline, incontinence, and orthostatic hypotension are a few examples.

PD is a growing global health concern, as evidenced by data from the Global Burden of Disease Survey. In 2017 alone, there were 1.02 million new cases of PD were reported,^[2]^ and as of 2019, it is estimated that almost 8.5 million people worldwide are living with the disease. PD surpassed all other neurological conditions in terms of both disability and fatalities. According to the latest estimates, PD caused 329,000 fatalities in 2019, an increase of over 100% since 2000. The disease is also responsible for 5.8 million disability-adjusted life years, marking an 81% increase since 2000.^[3]^

The current mainstay in managing PD is pharmacotherapy consisting of L-Dopa, an anchor drug. L-dopa, which is often combined with carbidopa (a peripheral decarboxylase inhibitor), is available in many formulations. These include oral disintegrating tablets, immediate release, controlled release, continuous enteral solutions, and inhaled powders, each of which has its pharmacokinetic profiles and attributes. In addition, other drugs include COMT (Catechol-O-methyltransferase) inhibitors (Tolcapone, Entacapone), MAO-B inhibitors (selegiline), dopamine agonists(bromocriptine), dopamine facilitators (amantadine), and anticholinergics(trihexyphenidyl).

Deep brain stimulation (DBS) is a surgical procedure that involves implanting an electrode into specific brain regions to generate electrical impulses that control abnormal brain activity and adjust for chemical imbalances, thus making it an effective treatment for a range of neurological conditions. The discovery of the subthalamic nucleus (STN) as a target for DBS in 1993 marked the beginning of its success as a treatment for PD. DBS has replaced ablative surgery such as pallidotomy as the standard treatment for refractory PD.

Patients with idiopathic PD with a great L-dopa response but motor difficulties due to long-term pharmacological treatment are ideal candidates for DBS.^[4]^ DBS has been shown to reduce the severity of both motor and non-motor symptoms in patients with PD, but it has not been shown to slow the progression of the disease.^[5]^ A retrospective analysis of veteran data revealed some moderate advantages in terms of improved life expectancy when comparing veterans who underwent DBS for PD with those who did not. However, it needs to be clarified whether this is solely due to the influence of DBS on the course of PD.^[6]^

Bibliometric analysis is a qualitative technique that examines the traits and trends of scientific publications within a given subject or discipline.^[7]^ Research collaborations, impact, and visibility can all be assessed using bibliometric analysis, along with research trends and gaps.^[8]^ In this study, we performed a bibliometric analysis to provide valuable insights into the research trends, patterns, and impact of scholarly works on DBS in Parkinson disease. Our central objective was to understand the current landscape of the most impactful research in DBS for PD, and how this has evolved over time. Our analysis of publication outputs, citation patterns, and collaboration networks will serve to map the existing knowledge landscape in the field. This, in turn, will help researchers, clinicians, and policymakers grasp the current state of research, identify key contributors, track trends, and make informed decisions.

## 2. Methodology

### 2.1. Database and search strategy

A systematic literature search was conducted using the Scopus database on March 13th, 2023. Due to its broad coverage of abstracts and citations, Scopus was selected as the primary database, as it is currently the largest database of scientific literature available. Web of Science is limited in terms of journal coverage, making it less practical to conduct an in-depth citation analysis. On the other hand, while PubMed permits more search terms to be used simultaneously, it does not have any citation analysis capabilities.^[9,10]^ An advanced search strategy was employed using the following keywords as main search terms: “Parkinson’s disease,” “Parkinson’s disease,” “PD,” “Parkinsonism,” “Deep brain stimulation,” and “DBS.” The search query used to obtain the results was: (TITLE-ABS-KEY (“Parkinson’s disease” OR “Parkinson’s disease” OR “PD” OR “Parkinsonism”) AND TITLE-ABS-KEY (“Deep brain stimulation” OR “DBS”)) AND (LIMIT-TO (DOCTYPE, “ar”)). Only published articles were included in this analysis and no time restrictions were applied. The search results were sorted in descending order based on their citation counts, and the first 1000 most cited publications were included in this analysis.

### 2.2. Data analysis and visualization

**Figure 1.**
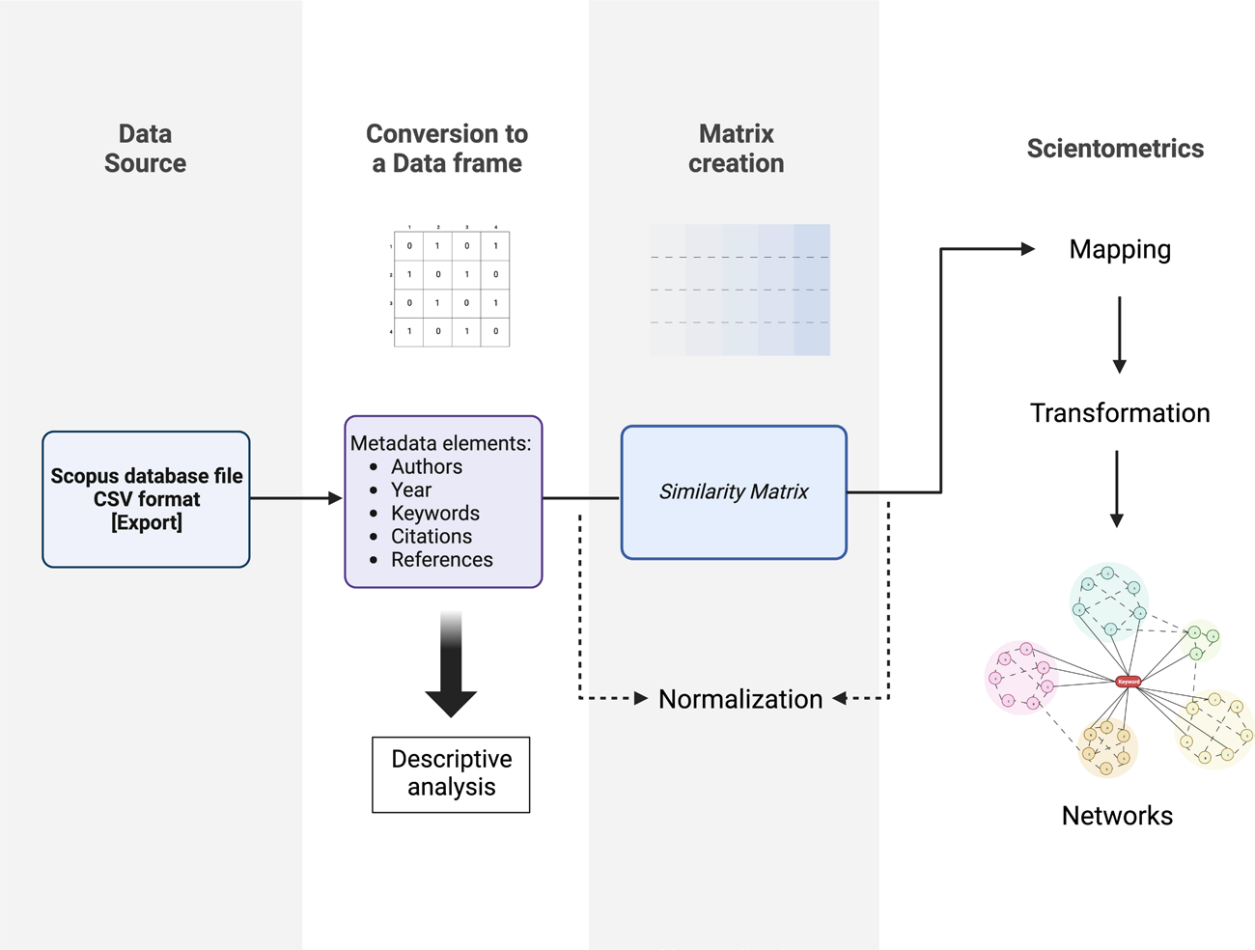
Workflow illustrating the scientometric analysis process.

The bibliometric analysis was conducted using the VOS Viewer Software (Version 1.6.19, Leiden University, Leiden, Netherlands) and the ‘Bibliometrix’ package in R Studio (Version 2022.12.0 + 353). Biblioshiny, a web interface for bibliometrix, was used for visualization. The metadata of the top 1000 cited publications in the field of DBS in Parkinson disease were exported as a CSV file from Scopus. This served as the source file input for software analysis. The workflow of the analysis is shown in Figure 1. An R data frame was then created by the software, which was used for descriptive analysis, and a similarity matrix was created by normalizing the co-occurrence data using association strength as a measure of similarity. A two-dimensional map was subsequently generated and subjected to transformation procedures, including translation, rotation, and reflection, resulting in the creation of network maps. The entire process was automated by the software.

We conducted a descriptive analysis using Bibliometrix, including publication characteristics, journal distribution, institutions, and countries. Additionally, we performed country-wise co-authorship and keyword co-occurrence analyses using VOS Viewer, which were further enhanced with overlay visualizations of the average publication years. This allowed us to assess the average publication year associated with specific keywords, terms, and sources. Co-occurrence indicates that the degree of relationship is determined by the number of documents in which they occur together. The unit of analysis for keyword analysis was “Author keywords,” which are terms chosen by the authors of the publication in scientific literature. The fractional counting method was used for all the analyses. Variations of words were standardized before keyword analysis, and words lacking meaning or utility were subsequently excluded from the analysis. Clusters were further identified and differentiated based on the different colors generated during the visualization. Qualitative analysis was then performed on these clusters to derive meaningful thematic areas. Subsequently, these thematic areas are discussed in the context of the existing literature to offer a comprehensive overview of the current state of research in the field. A trend analysis of the topics was also done, which analyzed the “Keywords Plus” which are keywords extracted from the titles of references cited in a paper. This facilitated a deeper understanding of the evolution of research topics in this field over time.

## 3. Results

### 3.1. Publication characteristics

We included the top 1000 cited articles in the analysis. The articles ranged from 1984 to 2021, and included 202 sources (journals, books, and conference reports). Each document has an average of 140.9 citations. In total, 31,854 references were included in this study. A total of 4898 keywords and 1337 authors’ keywords were analyzed. Table 1 presents an overview of the articles included in the analysis.

**Table 1:** Overview information of the articles analyzed.

Description	Results
Main information	
Timespan	1984:2021
Sources (journals, books, and conference papers)	202
Documents	1000
Document average age	14.9 yr
Average citations per doc*	140.9
References	31,854
Document contents	
Keywords plus (ID)†	4896
Author’s keywords (DE)‡	1337
Author information	
Authors	3749
Authors of single-authored docs	23
Authors collaboration	
Single-authored docs	25
Co-Authors per doc	7.47
International co-authorships %	30.1
Document types	
Article	1000

* Average citations per doc.: in our analysis, each document, on average, received 140.9 citations.

† Keywords plus (ID): additional keywords generated by the database to enhance search results.

‡ Author’s keywords (DE): keywords provided by the authors to describe their research.

**Figure 2.**
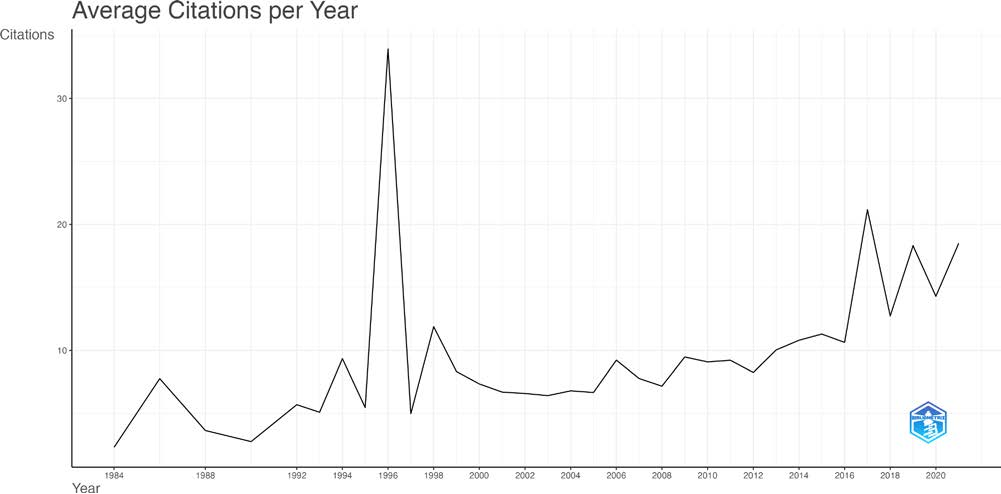
This figure illustrates the mean number of citations received per year by articles in the field of DBS for PD research. DBS = deep brain stimulation, PD = Parkinson disease.

Our analysis reveals that articles published in 1996 received the highest average number of citations per year, valued at 35.14. This was followed by articles published in 2021 and 2017, with average citation values of 24.6 and 24.19, respectively (Fig. 2). Mean citations per year for DBS in PD research are presented in Table S1, Supplemental Digital Content, http://links.lww.com/MD/M485 and Number of Documents and Authors, and Author Proportions in Table S2, Supplemental Digital Content, http://links.lww.com/MD/M486.

**Figure 3.**
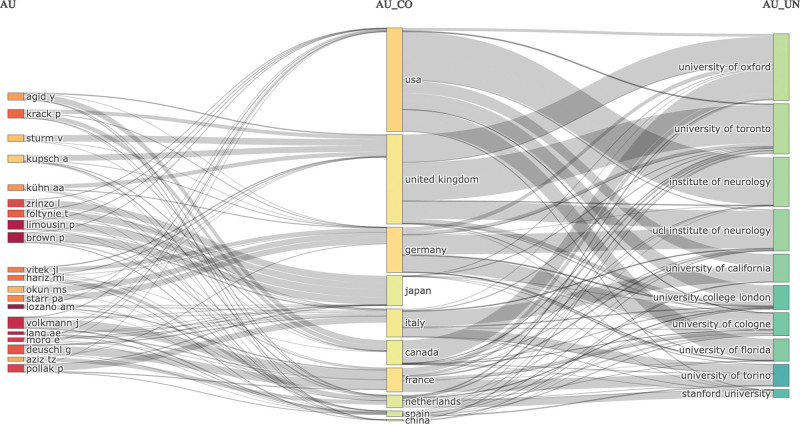
A tripartite network plot highlighting the collaborations among the top 10 authors, countries, and affiliations in DBS research for PD. DBS = deep brain stimulation, PD = Parkinson disease.

Figure 3 shows a three-field plot of the top 10 authors, countries, and affiliations. Most top authors have a strong relationship with the United Kingdom, followed by the USA, Germany, and Canada. The US institutions included the University of California, University of Florida, and Stanford University. The top authors in China had no relationship with US institutions but were linked to institutions in Canada and the United Kingdom. However, European countries have links to institutions in the USA, UK, and Canada.

### 3.2. Distribution of journals

A total of 202 different journals were obtained after processing the data of the 1000 most cited articles. The top 20 journals with the highest number of publications are listed in Table 2. The highest number of papers was from Movement Disorders (n = 98), followed by Brain (n = 78), Neurology (n = 65), and the Journal of Neurology, Neurosurgery, and Psychiatry (n = 64). Notably, JAMA Neurology stood out with the highest impact factor of 29.91 among the top 20 journals, further underlining its prominence in the field. JAMA Neurology published 15 articles on the subject, which, when considered in conjunction with its remarkable impact factor, signifies its substantial influence on DBS in PD research. Following closely in terms of impact factor were Brain (15.255), Journal of Neurology, Neurosurgery, and Psychiatry (13.65), and Neurology (12.258). The Journal of Neuroscience had the highest H-index, with a score of 471.

**Table 2:** Top 20 Journals with most DBS in PD articles.

Sources	Articles, n	H-index	Impact factor (2021)
Movement Disorders	98	209	9.698
Brain	78	351	15.255
Neurology	65	378	12.258
Journal of Neurology, Neurosurgery and Psychiatry	64	216	13.65
Journal of Neurosurgery	58	219	5.526
Experimental Neurology	32	195	5.62
Journal of Neuroscience	31	471	6.709
Parkinsonism and Related Disorders	30	105	4.402
Neurosurgery	29	207	5.315
Neuroimage	27	381	7.4
Annals of Neurology	21	308	11.274
Journal of Neurology	17	144	6.682
JAMA Neurology	15	243	29.91
European Journal of Neuroscience	15	213	3.698
Stereotactic and Functional Neurosurgery	15	66	1.643
Journal of Neurophysiology	13	252	2.714
Clinical Neurophysiology	11	191	4.861
Neurobiology of Disease	11	175	7.046
PLOS One	11	367	3.752
Brain Stimulation	10	89	9.611

DBS = deep brain stimulation, PD = Parkinson disease.

### 3.3. Distribution of institutions

The authors of the selected 1000 papers provided a total of 1079 affiliations with institutions, the top 20 of which are presented in Table 3. The University of Oxford was the most frequently mentioned institution among the top 20 with 145 reports, followed by the University of Toronto with 143, the Institute of Neurology, University College London with 111, the UCL Institute of Neurology with 97, and the University of California with 93.

**Table 3:** Top 20 institutions for DBS in PD research.

Affiliation	Country	Articles (n)
University of Oxford	United Kingdom	145
University of Toronto	Canada	143
Institute of Neurology, University College London	United Kingdom	111
UCL Institute of Neurology	United Kingdom	97
University of California	United States	93
University of Florida	United States	88
University of Cologne	Germany	75
University College London	United Kingdom	74
Stanford University	United States	68
University of Torino	Italy	53
Emory University	United States	50
Mayo Clinic	United States	50
University Hospital Cologne	Germany	47
University of Minnesota	United States	47
Baylor College of Medicine	United States	43
University of Amsterdam	Netherlands	43
University of Turin	Italy	42
Centre d’Investigation Clinique	France	39
Joseph Fourier University	France	38
Oregon Health and Science University	United States	38

DBS = deep brain stimulation, PD = Parkinson disease.

### 3.4. Distribution of countries/regions: exploring the global landscape

**Figure 4.**
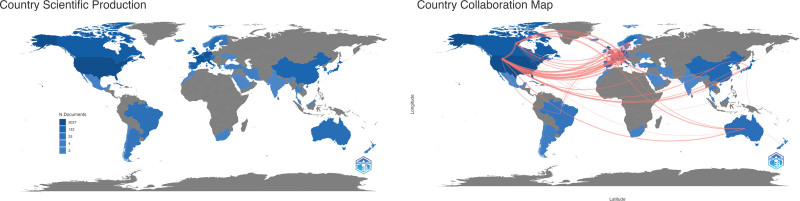
(Left) A world map visually represents the number of author appearances from different countries in the included articles, offering insight into each country’s scientific production. Darker blue shades indicate higher productivity, lighter shades represent lower productivity, and gray indicates no documented author appearances. (Right) Presents a world map illustrating author collaborations, showing international cooperation in DBS research. DBS = deep brain stimulation.

Figure 4 (left) is a world map representing the number of author appearances from a country in the included articles as an indicator of the country’s scientific production, depicted by the shades of color. The darker shades of blue signify relatively higher productivity and the lighter shades of blue signify relatively lower productivity, measured by the number of documents published in the literature. The gray color indicates the absence of documents.

**Figure 5.**
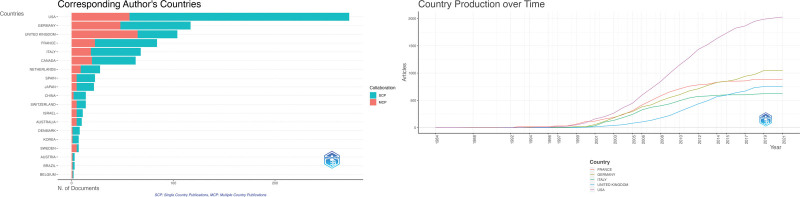
(Left) Displays trends in countries’ research output over the years, highlighting the chronological progression of articles associated with specific countries. (Right) Provides a graphical representation of the countries’ research production over time, demonstrating the growth in articles associated with each country based on the corresponding author’s affiliation.

Figure 4 (left) illustrates the trends in countries’ production over time. The first article was published in Germany in 1984. The first article in the USA was published in 1986, and since then, the number of articles published by the US has increased significantly compared to other countries. A similar trend can be observed in Figure 4 (right), which graphically represents the number of articles associated with a country, based on the corresponding author’s affiliation. The USA had 273 articles, Germany had 117 articles, and the United Kingdom had 104 articles. The multiple country publication (MCP) ratios, which represent the proportion of documents affiliated with a different country other than the corresponding author, are also shown in Figure 5 (right) and Table 4. The results showed that Sweden had the highest MCP ratio (0.714), closely followed by the United (0.625). Australia, Belgium, and Finland shared the third-highest MCP ratio of 0.5, indicating a relatively high probability of future collaboration. On the other hand, Lebanon, Argentina, the Czech Republic, Greece, and Austria did not appear to have a significant collaborative history in this particular research area, as their MCP ratio was found to be 0.

**Table 4:** Publication analysis of authors by Country: MCP ratio, citations, and articles.

Country	Number of author appearances, n	Articles, n	MCP Ratio	Total citations, n	Average citations/article, Mean
USA	2027	273	0.209	36,637	134.20
Germany	1045	117	0.41	16,567	141.60
United Kingdom	753	104	0.625	14,676	141.10
France	885	84	0.274	11,772	140.10
Italy	622	68	0.279	7726	113.60
Canada	394	63	0.317	10,091	160.20
Netherlands	215	28	0.321	4495	160.50
Spain	215	23	0.217	5158	224.30
Japan	216	22	0.227	2021	91.90
China	136	14	0.143	1453	103.80
Switzerland	116	14	0.357	2423	173.10
Israel	57	11	0.455	1497	136.10
Australia	53	10	0.5	995	99.50
Denmark	31	8	0.125	784	98.00
Korea	52	7	0.143	664	94.90
Sweden	64	7	0.714	526	75.10
Austria	55	3	0.333	1537	512.30
Brazil	37	3	0.333	301	100.30
Belgium	13	2	0.5	166	83.00

DBS = deep brain stimulation, MCP = multiple country publications, PD = Parkinson disease.

### 3.5. Key Research studies in DBS for Parkinson disease

The most cited paper in this field is from Deuschl et al, published in 2006 in *The New England Journal of Medicine* entitled “A Randomized Trial of Deep-Brain Stimulation for Parkinson Disease” with a total citation count of 2098, cited on average 116.56 times per year.^[11]^ Other highly cited papers in this field include research on the effectiveness of DBS on the STN or the globus pallidus interna (GPi), such as the studies by Obeso et al and Follett et al, both published in *The New England Journal of Medicine*.^[12,13]^ The paper by Poewe W et al titled “Parkinson Disease” published in *Nature Reviews Disease Primers* in 2017 also received a high number of citations (1353), focusing on the overview of DBS.^[1]^ Gradinaru V et al used optogenetics and solid-state optics to investigate the mechanisms of DBS and identify circuit elements involved in its therapeutic effects in their highly cited (1178 total citations) 2010 study published in *Science*^[14]^ (Table 5).

**Table 5:** Top 20 most cited articles for DBS in PD research.

Author(s)	Title	Year of publication	Journal	Total citations (TC)	Total citations per year
Deuschl G^[11]^	A randomized trial of deep-brain stimulation for Parkinson’s disease	2006	The New England Journal of Medicine	2098	116.56
Obeso JA^[12]^	Deep-brain stimulation of the subthalamic nucleus or the pars interna of the globus pallidus in Parkinson’s disease	2001	The New England Journal of Medicine	1363	59.26
Poewe W^[1]^	Parkinson disease	2017	Nature Reviews Disease Primers	1353	193.29
Gradinaru V^[14]^	Optical deconstruction of parkinsonian neural circuitry	2009	Science	1178	78.53
Weaver FM^[6]^	Bilateral deep brain stimulation vs best medical therapy for patients with advanced Parkinson disease	2009	Journal of the American Medical Association	1147	76.47
Follett KA^[13]^	Pallidal versus subthalamic deep-brain stimulation for Parkinson’s disease	2010	The New England Journal of Medicine	991	70.79
Benabid AL^[15]^	Chronic electrical stimulation of the ventralis intermedius nucleus of the thalamus as a treatment of movement disorders	1996	Neurosurgery	984	35.14
Rodriguez-Oroz MC	Bilateral deep brain stimulation in Parkinson’s disease: a multicentre study with 4 years follow-up	2005	Brain	886	46.63
Schuurman PR	A comparison of continuous thalamic stimulation and thalamotomy for suppression of severe tremor	2000	The New England Journal of Medicine	860	35.83
Frank MJ	Hold your horses: impulsivity, deep brain stimulation, and medication in parkinsonism	2007	Science	811	47.71
Little S^[16]^	Adaptive deep brain stimulation in advanced Parkinson disease	2013	Annals of Neurology	769	69.91
Hashimoto T	Stimulation of the subthalamic nucleus changes the firing pattern of pallidal neurons	2003	The Journal of Neuroscience	705	33.57
Defer GL	Core assessment program for surgical interventional therapies in Parkinson’s disease (CAPSIT-PD)	1999	Movement Disorders	654	26.16
Stefani A^[17]^	Bilateral deep brain stimulation of the pedunculopontine and subthalamic nuclei in severe Parkinson’s disease	2007	Brain	636	37.41
Bejjani BP	Transient acute depression induced by high-frequency deep-brain stimulation	1999	The New England Journal of Medicine	575	23.00
Kühn AA^[18]^	Reduction in subthalamic 8–35 Hz oscillatory activity correlates with clinical improvement in Parkinson’s disease	2006	European Journal of Neuroscience	573	31.83
Kühn AA	High-frequency stimulation of the subthalamic nucleus suppresses oscillatory beta activity in patients with Parkinson’s disease in parallel with improvement in motor performance	2008	NeuroSci	568	35.50
Rosin B^[19]^	Closed-loop deep brain stimulation is superior in ameliorating parkinsonism	2011	Neuron	559	43.00
Kumar R	Double-blind evaluation of subthalamic nucleus deep brain stimulation in advanced Parkinson’s disease	1998	Neurology	558	21.46
Hutchison WD	Neurophysiological identification of the subthalamic nucleus in surgery for Parkinson’s disease	1998	Annals of Neurology	544	20.92

DBS = deep brain stimulation, PD = Parkinson disease.

### 3.6. Co-authorship analysis

**Figure 6.**
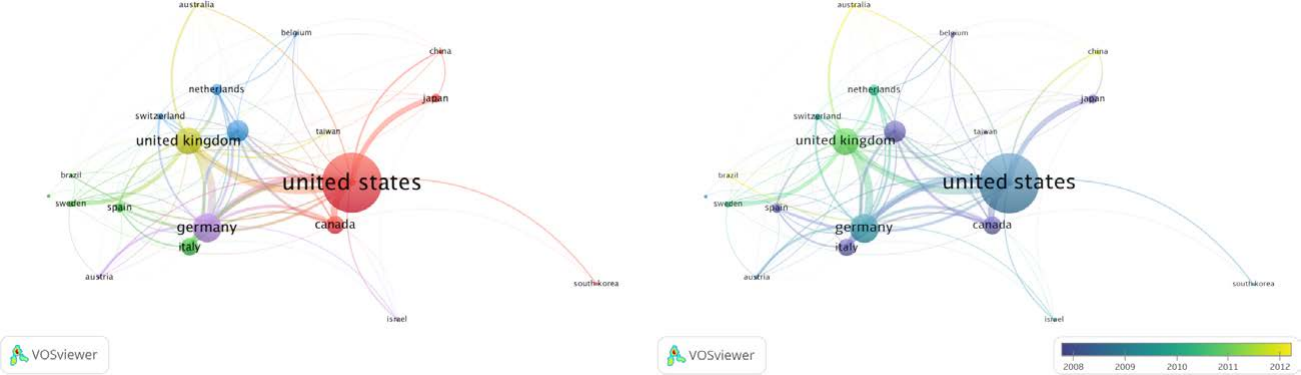
(Left) A network map of country-wise co-authorship analysis, highlighting 5 distinct clusters of countries engaged in DBS in PD research collaborations. The network visualizes the intensity of knowledge exchange among these countries. (Right) displays an overlay map of the same analysis, emphasizing collaboration patterns among countries in the field, notably highlighting recent networks involving Australia, Brazil, and China. DBS = deep brain stimulation, PD = Parkinson disease.

Country-wise co-authorship analysis of items related to DBS in PD revealed 5 clusters (Fig. 6, left). The largest cluster (Cluster 1-red) included Canada, China, Japan, South Korea, and the United States, indicating significant collaboration and knowledge exchange among these countries. Cluster 2 (green) included Brazil, Denmark, Italy, Spain, and Sweden, indicating a lower level of collaboration. Cluster 3 (blue) comprised Belgium, France, the Netherlands, and Switzerland, indicating moderate collaboration. Cluster 4 (yellow) and Cluster 5 (purple) were smaller, indicating relatively low levels of collaboration among Australia, Taiwan (China), the United Kingdom, Austria, Germany, and Israel. A temporal overlay reveals recent networks involving Australia, Brazil, and China.

The collaborative efforts of researchers studying the use of DBS in PD are shown in Figure 4 (right). The map displays the top 20 countries engaged in research collaboration, with the United States, Germany, and the United Kingdom being the top 3 collaborating countries. The lines between countries represent the frequency of collaboration between the researchers. This worldwide collaboration underscores researchers’ dedication to advancing knowledge and treatment of PD using DBS.

### 3.7. Distribution of keyword clusters

Keyword analysis forms an integral part of bibliometric analysis, allowing us to identify the trend of research hotspots over the years in different journals and to focus on newer emerging topics. Table 6 presents the top 25 author keywords with the highest frequency. These keywords included, “subthalamic nucleus” (250 publications) as the most common, followed by “basal ganglia” (77 publications), and “globus pallidus” (32 publications). “Middle-aged” appeared in 639 publications, while “local field potentials” and “tremor” were present in 28 and 31 publications, respectively.

**Table 6:** Top 25 most reported Author’s keywords for DBS in PD articles.

SR no.	Words	Occurrences, n
1	subthalamic nucleus	250
2	basal ganglia	77
3	globus pallidus	32
	tremor	31
5	local field potentials	28
6	functional neurosurgery	22
7	dystonia	21
8	beta oscillations	19
9	pedunculopontine nucleus	19
10	essential tremor	18
11	cognition	17
12	levodopa	17
13	thalamus	17
14	dopamine	16
15	quality of life	16
16	gait	13
17	magnetic resonance imaging	13
18	movement disorders	13
19	oscillations	13
20	pallidotomy	13
21	depression	12
22	stereotactic surgery	12
23	bradykinesia	11
24	neuropsychology	10
25	stereotaxy	10

DBS = deep brain stimulation, PD = Parkinson disease.

**Figure 7.**
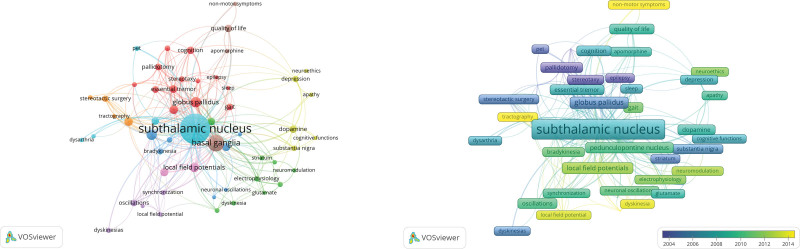
(Left) Presents a network map of keyword co-occurrence analysis, identifying 84 frequently occurring keywords grouped into 12 clusters. This map provides a comprehensive overview of keyword relationships and emerging research themes in DBS for the treatment of PD. (Right) Overlay map illustrating changes in keyword significance over time, shedding light on shifting research interests. DBS = deep brain stimulation, PD = Parkinson disease.

In total, we investigated 4896 keywords and 1337 author keywords from 1000 studies. To represent the relationships between the frequently occurring keywords, we generated a network map (Fig. 7 (Left)) that visualizes 84 keywords that appeared more than 5 times and were grouped into 9 clusters. This map provides a comprehensive overview of the co-occurrence of keywords, highlighting potential research themes and areas of interest in the field of DBS in PD.

These keywords were divided into 9 clusters: Neuropsychological Function (Cluster 1), Motor Circuit Electrophysiology (Cluster 2), Neuronal Rhythms and Tremor (Cluster 3), Psychological Aspects of PD (Cluster 4), Electrophysiological Synchronization (Cluster 5), Speech and Medication Effects (Cluster 6), Surgical Techniques and Imaging (Cluster 7), Symptoms and Quality of Life (Cluster 8), and Electrophysiological Metrics (Cluster 9).

The most commonly occurring keywords, as shown in Figure 7 and Table 6, were the subthalamic nucleus from Cluster 6, basal ganglia from Cluster 8, globus pallidus from Cluster 1, and Local Field Potentials from Cluster 9. This is shown in Figure 7 (density visualization map). Different colors represent the frequency of the occurrence of keywords, and yellow represents the most commonly occurring terms.

Figure 7 (Right) shows the overlay visualization. “DBS,” “Parkinson disease,” and “subthalamic nucleus” were the most frequently searched keywords, but their significance has been waning over the years. Most neurosurgical procedures, such as “pallidotomy,” “stereotaxic surgery,” and “neurosurgery,” are represented by darker colors and predominated in the earlier years of our analysis, indicating a shift from highly invasive neurosurgical procedures to DBS. In recent years, there has been a growing emphasis on terms such as “beta oscillations,” “local field potentials,” “beta activity” and “neuroethics,” indicating a shift of interest toward underlying pathophysiology and underlying mechanisms.

### 3.8. Distribution of trend topics and overlay visualization

**Figure 8.**
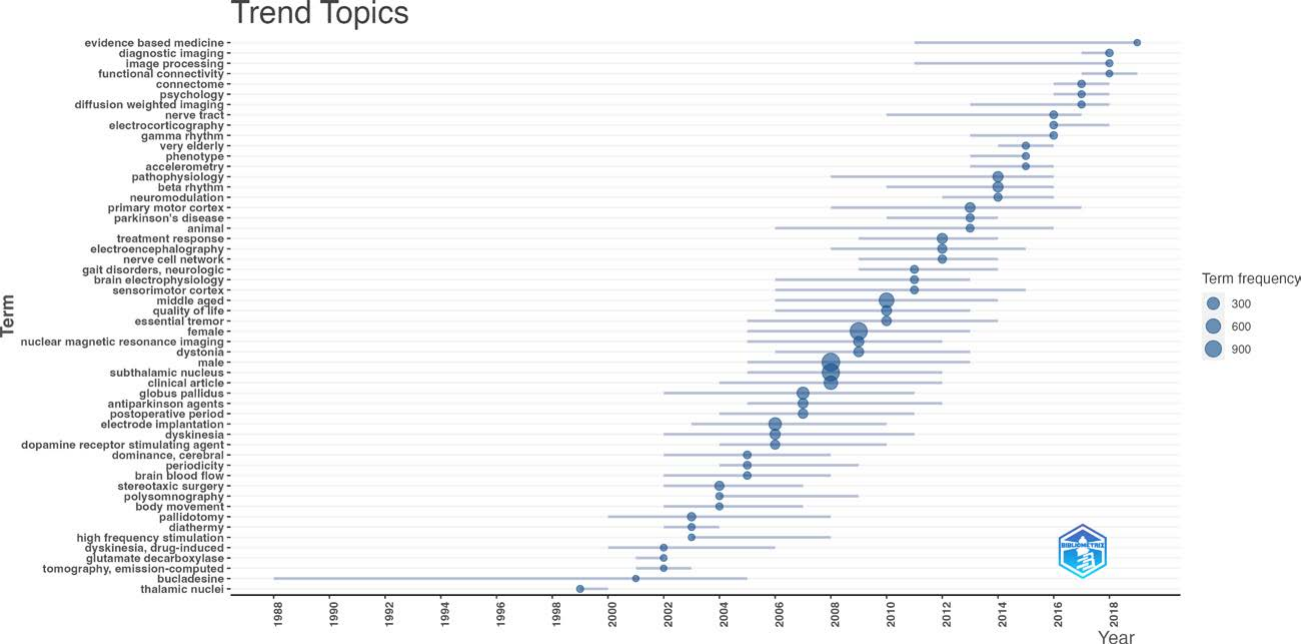
Chronological trend graphically depicting the frequency of trending research topics in DBS for PD. The size of each circle corresponds to the frequency of each topic. This visual representation showcases the evolution of research interest over time, from early topics to recent trends. DBS = deep brain stimulation, PD = Parkinson disease.

Trend topics were identified by analyzing “Keywords Plus” – keywords extracted from the titles of cited references. Figure 8 graphically represents the chronological trend in the frequency of the trend topics. Early trend topics included “thalamic nuclei” from 1999 to 2000. “Pallidotomy,” “diathermy,” “stereotaxic surgery,” and “electrostimulation” were seen to trend between 2000 and 2008. The globus pallidus” and “subthalamic nuclei” were seen from 2005 to 2011. The highest frequency, corresponding to the size of the circles, was observed in the “subthalamic nucleus,” “male,” and “female.” The topics that have gained maximum traction in recent years include “evidence-based medicine,” “diagnostic imaging,” “clinical outcomes,” “image processing,” “image processing,” “procedures,” “primary motor cortex,” and “connectome.” A shift in trend topics was noted from the early 2000s from mostly older methods (pallidotomy and stereotaxic surgery) and less emphasis on the disease process to later years, when the focus was placed on disease progression and mechanisms.

## 4. Discussion

In our systematic literature search within the Scopus database, we identified the most highly cited articles concerning DBS in PD, spanning a period from 1984 to 2021. These articles provide valuable insights and have made a significant impact on the field. The USA, Germany, and the United Kingdom have emerged as the foremost contributors to this field. Collaboration among countries is critical to advancing research in this domain. Three-field plot analysis showed that the top authors in the field were predominantly from institutions in the United Kingdom, including the University of Oxford, the Institute of Neurology, the UCL Institute of Neurology, and University College London. We identified 5 geographical clusters of top authors in the field, offering valuable insights into collaboration patterns and author relationships. The latest collaborative networks have emerged in countries such as Australia, China, and Brazil. These insights highlight the importance of international collaboration and suggest opportunities for enhancing cooperation among regions with fewer interactions. In the observed spike in average citations per year figure (Fig. 2), notably in the year 1996, it is important to highlight that this surge is attributed to a singular publication on “Chronic electrical stimulation of the ventralis intermedius nucleus of the thalamus as a treatment of movement disorders.” Only one paper published in that specific year has led to an inflation in the average citations, emphasizing the impact of this solitary contribution on the overall citation metrics. The paper contributed by establishing high-frequency VIM stimulation as a pivotal and reversible therapeutic approach for tremor in Parkinson disease, emphasizing precision in electrode placement and posing intriguing questions about its mechanisms.

In our analysis, we identified key journals that are central to the discussion on DBS in PD that have produced a substantial number of highly cited papers. Notable journals in this regard include Movement Disorders, Brain, Neurology, and the Journal of Neurology, Neurosurgery, and Psychiatry. It is worth noting that JAMA Neurology has the highest impact factor among these journals, whereas the Journal of Neuroscience has the highest H-index among the top 20 journals. This emphasizes their substantial contribution to the field by consistently publishing impactful work in the field.

### 4.1. Evolution of research hotspots

Our keyword cluster analysis revealed 9 distinct clusters, each representing a facet of DBS research in the context of PD. These clusters, prominently incorporating key terms such as “Globus Pallidus,” “Subthalamic Nucleus,” “Basal Ganglia,” and “Local Field Potentials,” Our discussion focuses on the 4 critical research areas drawn from these clusters.

1. DBS and Neuronal Oscillations in Parkinson Disease: In focal point, we delve into the role of DBS in modulating neuronal oscillations, with a particular focus on the Globus Pallidus.
2. Epilepsy, Sleep, and Gait in Subthalamic Nucleus Stimulation: The Subthalamic Nucleus, highlighted in Cluster 6, is a critical area of interest. We explore the effects of DBS on epilepsy, sleep patterns, and gait in patients who underwent stimulation of the subthalamic nucleus.
3. Complications of DBS in Parkinson Disease: In this focal point, we aim to address complications that may arise during DBS for Parkinson Disease.
4. Identification of brain targets for DBS: Cluster 9 underscores the importance of Local Field Potentials in pinpointing optimal brain targets for DBS. We explore the significance of precise brain target identification and its potential to enhance the effectiveness of DBS in the treatment of PD.

These thematic focal points collectively represent core areas of focus in DBS research. The subsequent discussion will provide a comprehensive understanding of the current status and potential implications for future research.

### 4.2. DBS and neuronal oscillations in Parkinson disease: current understanding and future directions

In recent years, there has been growing interest in investigating the relationship between DBS and neuronal oscillations in PD, resulting in a critical hotspot in PD research. This hotspot focuses on the role of DBS in modulating neuronal oscillations, and its potential clinical utility.

Extensive research has been conducted to investigate the impact of DBS on different types of neuronal oscillations, such as beta and gamma oscillations, which are frequently disrupted in PD.^[20]^ These studies have reported that DBS can effectively inhibit pathological beta oscillations and enhance gamma oscillations in the basal ganglia, resulting in an improvement in motor symptoms and other PD-related impairments.^[18,20,21]^

Despite the challenges in comprehensively understanding the underlying mechanisms of the effects of DBS on neuronal oscillations, the potential clinical utility of this technique in modulating these oscillations is promising. DBS has been shown to provide long-term benefits in improving motor symptoms, enhancing the quality of life, and reducing medication requirements in patients with PD. Furthermore, recent studies have explored the potential of closed-loop DBS, which can personalize therapy by modulating DBS based on the patient’s neural activity.^[18,20,21]^

Thus, despite the current gaps in understanding the exact mechanisms of DBS on neuronal oscillations, the clinical potential of DBS in improving motor symptoms, reducing medication requirements, and enhancing the quality of life of patients with PD is promising.

### 4.3. Epilepsy, sleep, and gait in subthalamic nucleus stimulation: current challenges and future directions

In recent years, the focus of PD research has shifted from solely treating motor symptoms to addressing non-motor symptoms that significantly affect patients’ quality of life. Among the identified research hotspots in this area is the effect of DBS of the subthalamic nucleus (STN-DBS) on non-motor symptoms such as epilepsy, gait, and sleep. While current medical and surgical treatments for PD are effective in treating motor manifestations, they are unable to completely treat non-motor symptoms, which has a significant impact on patients’ overall well-being.^[22]^ Thus, research efforts have shifted towards identifying effective treatment options for non-motor symptoms, including exploring the potential benefits of STN-DBS on non-motor symptoms in PD patients.

STN-DBS shows promise in treating refractory epilepsy, with underlying neuronal circuits between the cortex, thalamus, and basal ganglia thought to be responsible.^[23]^ Studies indicate that the subthalamic nucleus plays a role in clonic seizure activity,^[22,23]^ with EEG findings supporting this, but lacking definitive evidence.^[24,25]^ However, there is a need for research concerning the involved mechanisms and approaches, which could open new frontiers and offer numerous benefits to the affected patient population.

Several studies have found that sleep quality significantly improves in patients with Parkinson disease post-STN-DBS, positively affecting their quality of life.^[24,25]^ Additionally, the postoperative requirement for medication was found to decrease, motor symptoms improved, and depressive symptoms improved independent of the improvement in sleep quality.^[26]^ The pedunculopontine nucleus, a part of the reticular ascending system, was also found to be a good target for improving sleep disorders and STN, as observed in a study by Peppe et al.^[27]^

Although STN-DBS has shown promising results in treating motor symptoms of PD, non-motor symptoms, such as sleep disorders and gait abnormalities, still present a challenge to manage. Further studies are needed to determine the long-term benefits of STN-DBS for sleep disorders. STN-DBS alone may not be sufficient for levodopa-resistant gait abnormalities, and the pedunculopontine nucleus (PPN) is currently being explored as a potential target for treating these disturbances.^[28]^ However, additional studies are needed to establish the clinical effectiveness of this approach. Therefore, gait dysfunction remains a significant challenge in the management of PD.

### 4.4. Complications of DBS in Parkinson disease: mitigating risks and optimizing outcomes

While DBS has shown to be effective in managing PD symptoms, it can also be associated with various complications. These include hardware-related complications, such as infection, malfunction, lead migration, misplacement, stimulation-induced side effects, and cognitive and psychiatric adverse events. Therefore, research efforts have been directed towards identifying and mitigating these risks.^[29]^ This research hotspot aims to optimize outcomes for patients undergoing DBS therapy by improving surgical techniques, developing better hardware, and refining stimulation parameters. Additionally, it also focuses on developing predictive models for identifying patients who are at a higher risk of developing complications associated with DBS therapy, which could aid in personalized treatment planning.^[30]^

Recent studies have highlighted the importance of careful patient selection, appropriate surgical techniques, and optimal programming strategies to minimize complications and maximize the benefits of DBS therapy in patients with PD. Furthermore, advances in DBS technology have shown promise in improving the safety and efficacy of DBS therapies.^[31]^ The utility of this hotspot lies in its potential to improve patient outcomes and quality of life by reducing the complications associated with DBS therapy. It also highlights the need for individualized and multidisciplinary approaches to avoid complications in DBS therapy involving neurologists, neurosurgeons, psychiatrists, and neuropsychologists.^[29]^

Further research in this area, along with the development of innovative approaches and technologies, could lead to improved outcomes and reduced risks for patients with PD undergoing DBS therapy.

### 4.5. Identification of brain targets for DBS

Identifying the targets for DBS is a critical aspect of both therapeutic considerations and research in this field. In our study, we identified 4 main targets of DBS: the subthalamic nucleus, globus pallidus, pedunculopontine nucleus, and thalamus.

Before the advent of DBS, neurosurgical interventions were the primary methods for managing refractory Parkinson disease. The first form of DBS that gained widespread acceptance was thalamic DBS. It was found to be particularly effective in controlling tremors associated with both Parkinson disease and essential tremors. The most common target for thalamic DBS is the ventral intermediate nucleus, especially in patients with tremors as their only significant issue.^[15,32]^

Currently, the globus pallidus and subthalamic nucleus are the primary targets of DBS. DBS-GPi was found to improve dyskinesias and on–off fluctuations and was shown to have a relatively safe profile.^[33]^ However, there has been a shift to the subthalamic nucleus as a target for DBS, given the immediate benefit and improved long-term outcomes provided for Parkinsonian symptoms in animal and human studies. Postoperative motor fluctuations, tremors, and the need for anti-parkinsonian medications were significantly reduced.^[34,35]^ However, there is still controversy regarding which of the 2 DBS methods, targeting the STN or GPi, is superior. While some studies argue in favor of the STN-GPi as a better approach, others report no significant differences between the 2 methods.^[13,36]^

In addition to the traditional targets for DBS, there is growing interest in exploring newer targets such as the rostral zona incerta, substantia nigra pars reticulata, parafascicular nucleus of the thalamus, nucleus basalis of Meynert, and dentate nucleus of the cerebellum.^[34]^ However, establishing these as reliable therapeutic options requires large-scale studies with larger patient cohorts.

One such potential target is the PPN, located in the mesencephalic locomotor region. It has been hypothesized to be involved in several PD symptoms that are resistant to levodopa treatment, including sleep disorders, gait freezing, and loss of balance. These hypotheses are further supported by the findings of Jellinger,^[35]^ who conducted a postmortem study on individuals with PD. The study revealed that the degree of degeneration in the PPN was directly correlated with the severity of gait dysfunction prior to death. Therefore, targeting the PPN with DBS could potentially positively influence gait dysfunction associated with PD, and there is also evidence to suggest that low-frequency bilateral PPN-DBS can improve gait and balance in PD patients. A case report documented improvements in gait and balance assessments in 2 patients with PD who underwent PPN-DBS.^[37]^ This approach was found to be effective in improving bradykinesia, rigidity, and gait disorders, although it did not have a significant effect on tremors.^[37]^ This highlights the potential need for PPN-STN stimulation. This has been explored in a study by Stefani et al, who found that an intervention consisting of L-dopa with combined stimulation of the PPN and STN led to significant performance gains in patients with advanced PD.^[17]^ However, the specific benefits of this type of stimulation and potential adverse effects of PPN-DBS have not been fully explored in detail. Additionally, there is a need for larger patient studies to draw more definitive conclusions on the inclusion criteria for potential patients and study design. Further research is necessary to better understand the potential benefits and risks of dual PPN-STN stimulation in PD. Neuroethics, focusing on ethical issues raised by our increased and constantly improving understanding of the brain, has also gained popularity because it is essential to consider ethical implications, especially when discussing an invasive and expensive procedure such as DBS.

Animal studies are important for gaining insight into the mechanisms of DBS. In particular, newer DBS methods and adaptive DBS have played a major role in furthering our understanding of DBS. A study by Wenger et al comprehensively analyzed the role of rodent models in assessing experimental DBS and found the unilateral 6-hydroxydopamine (6-OHDA) nigrostriatal lesion rat model to be the most common model for the same.^[38]^ Two animal studies comparing adaptive DBS and conventional DBS utilized MPTP-treated monkey models and found promising results for the former, significantly contributing to our understanding of closed-loop DBS.^[19,39]^ They have also improved our insights into DBS targeting, with significant potential to improve long-term patient outcomes.

## 5. Future frontiers

### 5.1. Personalized DBS targeting based on individual connectome mapping

While DBS is an effective therapy for patients with PD, the conventional targeting approach is not always optimal, and patients can still experience motor symptoms despite receiving therapy.^[40]^ Researchers have recently explored personalized approaches to DBS targeting based on individual connectome mapping using resting-state functional magnetic resonance imaging (rs-fMRI) data.^[41]^ The variability of rs-fMRI scans and different algorithms pose challenges for individualized connectome mapping.

To address this issue, several studies have proposed novel tools and frameworks for personalized DBS targeting in PD. For example, Loh et al developed the Tor-PD connectome to identify PD-specific functional networks using rs-fMRI data from 75 patients with PD.^[42]^ Hollunder et al^[43]^ proposed a personalized approach to DBS targeting that accounted for the variability in symptom profiles and comorbidities among patients by assigning personalized weights to different symptom networks. Bonmassar and Makris^[44]^ proposed using high spatial resolution connectome data, personalized DBS targeting based on individual anatomical differences, and atlases with estimates of inter-subject variability to improve DBS neurosurgery targeting.

Several studies have proposed data-driven algorithms for optimal stimulation parameters in patients with PD undergoing DBS. Roediger et al^[45]^ evaluated the effectiveness of a data-driven algorithm, StimFit, in suggesting optimal stimulation parameters based on electrode location. Chen et al^[46]^ developed a personalized whole-brain modeling approach that simulates the effects of focal stimulation on brain networks in a patient-specific manner.

In addition, there has been a recent rise in academic interest in the concept of closed-loop adaptive DBS (aDBS) owing to the various challenges encountered with conventional DBS (cDBS) in the form of side effects and the need for constant monitoring. Several studies have conducted comparisons and have found promising results for the former. Little et al^[16]^ reported improved motor scores in cases of both aDBS and cDBS, with significant improvement in the former in comparison. In a similar vein, other studies also found that aDBS had beneficial effects such as better symptomatic control of dyskinesias, gait, tremors, and speech problems, and had reduced mean total energy delivered when compared to its conventional counterpart.^[47–49]^

These studies present a promising opportunity for developing a personalized approach to DBS targeting patients with PD based on individual connectome mapping, improved understanding of the effects of DBS on brain connectivity, and data-driven algorithms for optimal stimulation parameters. However, further research is required to validate the efficacy and safety of these personalized approaches before they can be widely implemented in clinical practice.

### 5.2. DBS integration with noninvasive neuromodulation technology

The field of DBS is rapidly evolving, with developments in electrode technology and control algorithms moving towards individualized therapy that tracks the clinical state.^[50]^ Additionally, the adoption of new technologies is anticipated to be supported by developments in technological trends, such as the “Internet of Things” and device miniaturization, which will enable more continuous patient assessments and more complex control of various timelines. This may include integrating data from peripheral sensors and using machine learning methods to aid decision-making.^[51]^ Advances in minimally invasive techniques, such as implantation through less-invasive vascular routes, may also reduce the invasive nature of DBS surgery.^[52]^ Looking further into the future, using optogenetics or other cell-based technologies, new methods can be developed to activate the nervous system. The capacity to modify and control brain circuits can be considerably improved using specific targeting methods.^[53]^ Furthermore, the function of glia and astrocytes in brain computing may offer an additional degree of flexibility in modifying neural circuit activity.^[54]^ Overall, the rate of technological development coupled with the significant burden of neurological disorders motivates innovations in integrating DBS with new and emerging noninvasive neuromodulation technologies to treat PD.

Recent research has shown promising neuroprotective and disease-modifying effects of noninvasive brain stimulation techniques, particularly transcranial direct current stimulation, in improving cognitive performance in PD.^[55]^ These effects are believed to be mediated through multiple mechanisms, including the modulation of excitability, lysosomal and non-lysosomal pathways, inflammatory responses, and interference with BBB permeability.^[56–59]^ The potential for integrating DBS with new and emerging noninvasive neuromodulation techniques, such as noninvasive DBS, is an exciting area of research that could provide selective and precise targeting of the deep brain regions primarily implicated in neurological pathologies, with a substantial clinical impact.^[60]^ Further research is needed to explore the potential of combining DBS with noninvasive neuromodulation techniques for treating PD and other proteinopathies.

Noninvasive brain stimulation (NIBS) techniques, such as transcranial magnetic stimulation and transcranial current stimulation, are promising alternative therapies for PD because of their safety, portability, and personalization potential.^[61]^ However, the efficacy of NIBS remains to be confirmed in large multicenter randomized controlled trials, and more powerful stimulation protocols are needed to improve patient quality of life.^[62]^ Three modes of clinical applicability have been proposed for NIBS: intermittent application, potentiation of combined interventions, and chronic stimulation, as with DBS, but this is only feasible with transcranial current stimulation and not repetitive transcranial magnetic stimulation.^[62]^ According to recent data, the long-term benefit following stimulation is relatively moderate, and the adjuvant effect of other treatments could be masked by a ceiling effect.^[62]^

Although NIBS techniques are considered safe, some side effects have been reported. Strategies to counter these side effects have emerged, such as topical anesthetics on the scalp or high-frequency amplitude-modulated stimulation.^[63,64]^ New paradigms are emerging and biomarkers have been uncovered to provide a new rationale for future assessment and treatment approaches. There has been increased interest in the function of oscillatory activity in cortico-basal ganglia loops in information gating, coupling, and decoupling of distal brain locations. New biomarkers that correlate with clinical outcomes are being sought.^[65]^ Although little research has been conducted on this topic, the nonlinearity of brain interactions may be a fruitful area for future studies.^[66]^ Further research is required to validate the effectiveness and safety of these approaches for treating PD and other proteinopathies. Integrating DBS with noninvasive neuromodulation techniques holds promise as an exciting area of research that could revolutionize the treatment of PD.

### 5.3. Strengths and limitations of our study

The analysis included articles from a reputable database, Scopus, which provides a comprehensive overview of the research landscape in DBS for PD and analysis was performed with the help of Bibliometrix.^[67]^ Bibliometric analysis provides an objective method to quantitatively assess the impact and significance of articles in the field. Furthermore, this analysis can provide valuable insights into the evolution of the most influential research in the field.

Additionally, in line with similar works in other fields, we conducted country-wise co-authorship and keyword co-occurrence analyses using VOS Viewer, which was used in similar works in other fields.^[68–71]^

This study has some limitations. Analyzing the top 1000 most-cited articles introduces an evident bias by excluding recent literature that may not have had adequate time to accumulate citations. This was done, to ensure the analysis of the most impactful and validated research in the field. An additional effort was made to review the recent literature on thematic areas identified through clusters in keyword analysis. Furthermore, the analysis may be limited by language bias, as it only considers articles in the English language.

## 6. Conclusion

Our bibliometric analysis of the top 1000 publications on DBS use in PD from 1984 to 2021 highlights the prominence of the UK and US in DBS research. The evolution of research hotspots reveals 9 clusters, emphasizing DBS and neuronal oscillations, STN stimulation’s impact on non-motor symptoms, DBS complications, and precise brain target identification. These areas are central to contemporary DBS research and hold the potential to redefine Parkinson treatment. Moreover, our analysis demonstrates a shifting landscape in neurosurgical procedures, transitioning from highly invasive approaches like “pallidotomy,” “stereotaxic surgery,” and “neurosurgery” to the more refined domain of DBS. In recent years, there has been a growing emphasis on terms such as “beta oscillations,” “local field potentials,” “beta activity,” and “neuroethics,” indicating an evolving interest in underlying pathophysiology and mechanisms. The pressing need for personalized DBS therapy, based on individual connectome mapping, is evident, as is the potential for integrating DBS with noninvasive neuromodulation techniques such as NIBS.

## Author contributions

**Conceptualization:** Vinay Suresh.

**Data curation:** Tirth Dave.

**Formal analysis:** Vinay Suresh.

**Resources:** Tirth Dave.

**Writing – original draft:** Vinay Suresh, Tirth Dave, Shankhaneel Ghosh, Rahul Jena, Vivek Sanker.

**Writing – review & editing:** Vinay Suresh, Tirth Dave, Shankhaneel Ghosh, Rahul Jena, Vivek Sanker.

## Supplementary Material
