# Supplementary material for: Deep brain stimulation in Parkinson’s disease: A scientometric and bibliometric analysis, trends, and research hotspots
Source: Medicine (Baltimore). 2024 May 17;103(20):e38152. doi: 10.1097/MD.0000000000038152 (PMC11098246; doi:10.1097/MD.0000000000038152)
Supplement: Supplementary file 1 [file medi-103-e38152-s001.docx]

Supplementary Table 1: Mean citations per year for DBS in PD research

| Year | Mean Total Citations per Year | Citable Years |
| --- | --- | --- |
| 1984 | 2.38 | 40 |
| 1986 | 7.97 | 38 |
| 1988 | 3.75 | 36 |
| 1990 | 2.85 | 34 |
| 1992 | 5.88 | 32 |
| 1993 | 5.27 | 31 |
| 1994 | 9.67 | 30 |
| 1995 | 5.66 | 29 |
| 1996 | 35.14 | 28 |
| 1997 | 5.17 | 27 |
| 1998 | 12.35 | 26 |
| 1999 | 8.66 | 25 |
| 2000 | 7.65 | 24 |
| 2001 | 7 | 23 |
| 2002 | 6.89 | 22 |
| 2003 | 6.73 | 21 |
| 2004 | 7.14 | 20 |
| 2005 | 7.02 | 19 |
| 2006 | 9.74 | 18 |
| 2007 | 8.23 | 17 |
| 2008 | 7.62 | 16 |
| 2009 | 10.11 | 15 |
| 2010 | 9.74 | 14 |
| 2011 | 9.93 | 13 |
| 2012 | 8.95 | 12 |
| 2013 | 10.95 | 11 |
| 2014 | 11.89 | 10 |
| 2015 | 12.56 | 9 |
| 2016 | 11.97 | 8 |
| 2017 | 24.19 | 7 |
| 2018 | 14.86 | 6 |
| 2019 | 21.99 | 5 |
| 2020 | 17.88 | 4 |
| 2021 | 24.67 | 3 |
