# Supplementary material for: Deep brain stimulation in Parkinson’s disease: A scientometric and bibliometric analysis, trends, and research hotspots
Source: Medicine (Baltimore). 2024 May 17;103(20):e38152. doi: 10.1097/MD.0000000000038152 (PMC11098246; doi:10.1097/MD.0000000000038152)
Supplement: Supplementary file 2 [file medi-103-e38152-s002.docx]

Supplementary Table 2: Number of Documents and Authors, and Author Proportions

| **Documents written** | **N. of Authors** | **Proportion of Authors** |
| --- | --- | --- |
| 1 | 2643 | 0.705 |
| 2 | 508 | 0.136 |
| 3 | 198 | 0.053 |
| 4 | 133 | 0.035 |
| 5 | 65 | 0.017 |
| 6 | 36 | 0.01 |
| 7 | 36 | 0.01 |
| 8 | 31 | 0.008 |
| 9 | 15 | 0.004 |
| 10 | 17 | 0.005 |
| 11 | 8 | 0.002 |
| 12 | 6 | 0.002 |
| 13 | 5 | 0.001 |
| 14 | 6 | 0.002 |
| 15 | 5 | 0.001 |
| 16 | 5 | 0.001 |
| 17 | 3 | 0.001 |
| 18 | 1 | 0 |
| 19 | 3 | 0.001 |
| 20 | 2 | 0.001 |
| 21 | 3 | 0.001 |
| 22 | 1 | 0 |
| 23 | 1 | 0 |
| 24 | 3 | 0.001 |
| 25 | 1 | 0 |
| 26 | 1 | 0 |
| 27 | 2 | 0.001 |
| 28 | 1 | 0 |
| 29 | 1 | 0 |
| 30 | 1 | 0 |
| 31 | 2 | 0.001 |
| 36 | 1 | 0 |
| 38 | 1 | 0 |
| 40 | 1 | 0 |
| 44 | 1 | 0 |
| 53 | 1 | 0 |
| 60 | 1 | 0 |
